# 

**Published:** 1883-06

**Authors:** 


					﻿JUNE, 1883.
Jtì+RTIETH YEAR.
IIAELS
JOÜ1IAL
OF
TTriro A T m'O’!
ttZaAlalM
' Guaranteed Circulation 200,000 Copies in 12'Months.
E. H. GIBBS, A.M., M.D., Editor,
No. 21 CLINTON PLACE, EIGHTH STREET, NEW Ï0BK.
> TERMS : $1 a Year.
9
Single number. 10 cts.
				

